# Does integration matter? an international cross-sectional study on the relationship between perceived public health and primary care integration and COVID-19 vaccination rates

**DOI:** 10.1371/journal.pone.0317970

**Published:** 2025-02-21

**Authors:** Sumeet Sodhi, Rifka Chamali, Devarsetty Praveen, Manushi Sharma, Marcelo Garcia Dieguez, Robert Mash, Felicity Goodyear-Smith, David Ponka

**Affiliations:** 1 Besrour Centre for Global Family Medicine, College of Family Physicians of Canada, Mississauga, Canada; 2 Toronto Western Hospital, University Health Network, Toronto, Canada; 3 Faculty of Medicine, Department of Family and Community Medicine, University of Toronto, Toronto, Canada; 4 The George Institute for Global Health India, New Delhi, India; 5 Faculty of Medicine, University of New South Wales, Sydney, Australia; 6 Prasanna School of Public Health, Manipal Academy of Higher Education, Mangaluru, India; 7 Centre for Study of Health Professions Education, Universidad Nacional del Sur, Bahia Blanca, Argentina; 8 Faculty of Medicine and Health Sciences, Department of Family and Emergency Medicine, Stellenbosch University, Stellenbosch, South Africa; 9 Department of General Practice and Primary Health Care, University of Auckland, Auckland, New Zealand; 10 Department of Family Medicine, University of Ottawa, Ottawa, Canada; Faculdade Arthur Sa Earp Neto Faculdade de Medicina de Petropolis, BRAZIL

## Abstract

**Background:**

Immunisation against COVID-19 is crucial for controlling the pandemic, yet global challenges persist in vaccine coverage and equitable distribution. A well-integrated primary health care approach can enhance vaccination programmes.

**Aim:**

To explore the relationship between perceived PC (primary care)-PH (public health) integration, as well as other vaccination program implementation factors, and national COVID-19 vaccination coverage.

**Design and setting:**

A convenience sample of self-identified primary care professionals completed an online survey on COVID-19 vaccination programme implementation and their perceptions of PC-PH integration.

**Methods:**

Countries with ≥5 responses were included in the data analysis. COVID-19 vaccination implementation approach and perceived PC-PH integration against COVID-19 vaccination coverage was investigated using bivariate and subgroup analyses, Spearman correlation, and linear regression.

**Results:**

A total of 394 responses from 32 countries were analysed. Participants included primary care providers, academics, and researchers. The median national COVID-19 vaccination coverage was 28.41% at time of study. Perceived barriers included patient hesitancy and vaccine supply shortages, while facilitators included vaccine product choices, equity, and community engagement. The study revealed a positive relationship between perceptions of PC-PH integration and national vaccination coverage in upper-middle and lower-middle income countries.

**Conclusion:**

Perceived PC-PH integration increased with decreasing economic quartiles and this perception was linked to actual national vaccination coverage. Integration may be especially important for countries with lesser vaccine supply. High-income countries may benefit from increased collaboration between PC and PH to enhance vaccination efficiency. The findings contribute to understanding the role of PC-PH integration in vaccination programmes in different settings.

## Introduction

Immunisation was a key factor in controlling the COVID-19 pandemic [1,2], however vaccine coverage and ensuring equitable distribution of vaccines between and within countries continues to be a challenge globally [3]. Local and regional challenges have included acceptability of the COVID-19 vaccine among developed countries, and affordability and accessibility of vaccines among developing countries [4]. A strong and integrated primary health care (PHC) approach can support delivery of successful vaccination programmes, and vice versa, successful vaccine programme delivery can support strengthening PHC [5].

The United Nations defines PHC as a whole-of-society approach to health that prioritizes people’s needs and preferences as early as possible along the continuum from health promotion and disease prevention to treatment, rehabilitation, and palliative care, and as close as is practical to people’s everyday environment [6]. In a PHC model of care, primary care (PC) and public health (PH) services have overlapping functions along the continuum described above, and can lead to greater health outcomes through the synergistic actions of coordination and integration [7,8]. The World Health Organization (WHO) has outlined four actions that could facilitate PC-PH integration: enabling PC to deliver more protective, promotive, and preventative services to a defined population; improving communication and coordination between PH authorities and PC providers and managers; sharing knowledge and data to evaluate the impact of both individual and population-focused services on health; and strengthening the surveillance function of PC and more effectively linking this to PH surveillance [8].

Evidence suggests that integrating first-contact PC services with community-oriented, population-wide PH approaches for implementation of non-COVID-19 immunisation programmes in the past has led to improved access and wider national coverage [7–10]. Primary care providers (PCPs) are often trusted sources for vaccine information, can better build local community trust, able to more easily reach vulnerable and marginalised communities, and can contribute overall to improved vaccination uptake [7,11–13].

In an integrated PHC model, PCPs deliver vaccines and are supported by PH in appraising the effectiveness and safety of vaccines, providing rapid response to outbreaks, and establishing vaccine schedules and vaccine policies [14]. This model of integrated PHC, where PC and PH work together (PC-PH integration) for implementation of a vaccination programme has proved successful in past vaccination campaigns for influenza [7,9,13,15], (including H1N1 [12]), human papilloma virus (HPV) [16], and meningococcal-B disease [17].

The potential positive benefit of the PCP role to support mass COVID-19 vaccination campaigns has been well-articulated [13,18], however, there is a gap in the evidence around the actual implementation approaches used in different PHC contexts to achieve this. In addition, PC-PH integration, especially in the context of COVID-19 national responses, has been varied and sometimes ill-defined. Both positive and negative experiences have been reported: negatives include bypassing the first-contact role of PC and lack of leadership, and positives include clear communication and rapid mobilisation of resources [19,20]. Insights have been emerging from different areas on the integration of primary care, public health and community-based organizations in the aftermath of the COVID-19 pandemic and vaccination efforts, and a call for action for integrated primary health care systems to address future pandemics across high, middle and low income countries has been highlighted [21]. Post-COVID-19 pandemic facilitators of vaccination campaigns in an integrated primary care system include: integrating immunization with existing health campaigns; leveraging trustworthy relationships between community and health workers; government support at various levels for service delivery and financing mechanisms; active community engagement; and a multisectoral approach to regulations and strageties. The major barriers identified have been the cost of integration and challenges with human resources for health including training; the lack of evidence around cost-effectiveness of integration has also been noted as a potential policy challenge [22,23].

This study builds on a previous international survey investigating the relationship between the perceived strength and role of a county’s PC system and COVID-19 mortality early in the pandemic [20]. While PC per se did not predict mortality, the study showed a positive effect of PC-PH integration. The aim of our current study was to explore the relationship between perceived PC-PH integration, as well as other vaccination program implementation factors, and national COVID-19 vaccination coverage.

## Methods

### Setting and participants

This was an online mixed-methods cross-sectional study using a convenience sample. The online survey instrument was developed and validated by the team based on study objectives and WHO frameworks for monitoring and evaluation of vaccination programmes, as well as evidence-based indicators for integration between PC and PH [19,24–27]. A formative committee developed the survey instrument, and a summative committee verified and validated content. The process of survey development has been previously published [28]. The survey was translated to all official United Nations languages (Arabic, Chinese, English, French, Russian, Spanish), plus Portuguese, and administered via Qualtrics XM. (Full survey available in S1 File).

The study population comprised self-identified primary care professionals globally. Participants were recruited via an invitation accompanied by a web link or QR code to the survey through email and/or online newsletters with member-based organisations, drawing on the researchers’ professional networks (S2 File). Recruitment was supplemented by snowballing, with participants encouraged to share the survey web link with colleagues, hence reporting the invitation denominator is not possible. The survey ran from the 31^st^ of October 2021 to 28^th^ of February 2022.

### Response variable

For this study, national COVID-19 vaccination coverage was defined as the share of the population who had completed an initial protocol of any COVID-19 vaccine (one or two doses depending on the vaccine product) as of October 31, 2021. Data were extracted from Our World in Data Coronavirus Pandemic online [3].

### Predictor variables

The survey asked about the implementation approach to COVID-19 vaccination programs, as well as perceptions on PC-PH integration. To assess the implementation approach, planning, coordination and service delivery were explored, including: type of COVID-19 vaccine used; vaccine registration processes and tracking; location of vaccine administration; cost of vaccination from the recipient’s perspective; access and equity for priority groups; barriers to implementation; and perceptions of effectiveness of vaccine programme strategies. For PC-PH integration, the role of PC as well as different attributes of a strong PHC approach was surveyed, including: the level and type of PC involvement (role, extent, site of vaccine administration, perspectives on best practices and suggestions for improvement); health system infrastructure (use of a unique patient identifier across health care settings, immunisation registries); strategy in place to assign patients to a PC facility; type and scope of care provided to the community; gatekeeper role of PC perceptions on degree of community engagement; and extent of PC-PH integration.

### Data analysis

Individual participant responses were aggregated to country level to perform bivariate analyses with country-level outcomes. For any country to be included, a minimum number of five responses was required, based on a perception of adequate representation at five responses from previous studies [20]. Subgroup analyses were performed to control for country income classification quartile (low, lower-middle, upper-middle and high) [29]. In addition to descriptive statistics, bivariate analyses were performed to determine the correlation between predictor and response variables, and Spearman correlation coefficients (*r*_*s*_) with p values were calculated for pairs of variables. In some cases, simple linear regression models were conducted to determine the strength of any associations between PC-PH integration and national vaccination coverage. For numeric responses (e.g. Likert scale), country-level responses were calculated as the mean across all participants in each country. For multiple-choice responses, country-level responses were calculated as the proportion of responders from each country who selected that response. For open-ended questions, responses were coded thematically and then calculated in the same manner as multiple-choice responses. Data were analysed in the Qualtrics XM analytic tool and Microsoft Excel for data cleaning and statistical analyses performed using SPSS (version 28).

### Ethics approval

Ethics approval was granted from the Health Sciences Research Ethics Board at the University of Toronto (Protocol #27873). Participants provided electronic consent by accessing the survey webpage. They were presented with consent details on the landing page, including study information, voluntary participation, risks and benefits, and confidentiality. Participants then had the choice to click "I agree" to confirm their consent and participation or "I do not agree" if they declined.

## Results

### Participant characteristics

**Fig 1:**
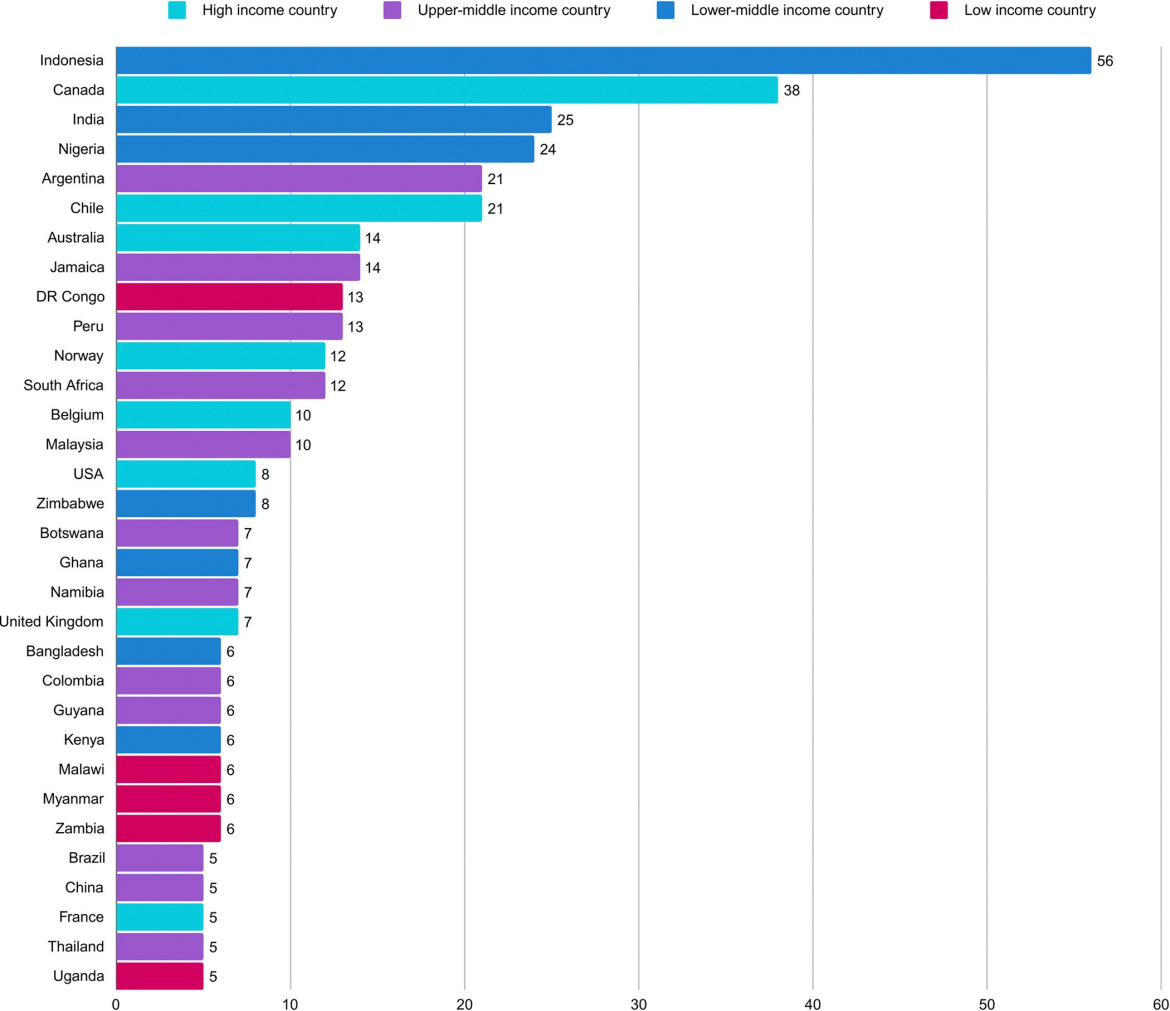
Respondents by country (for countries with ≥5 responses).

We received 477 responses across 84 countries. After excluding countries with <5 responses, there were 394 responses across 32 countries (Fig 1). Participants’ professional and personal characteristics are listed in Table 1. Most participants identified themselves as PCPs (50.0%) or academics/researchers (29.9%) and, the majority of PCPs identified as general practitioners or family doctors (91.9%). Just over half (53.0%) of participants identified as female with their mean years of professional experience 14 (range 0–48). The majority completed the survey in English (77.7%) but there were responses in all other languages except Russian and Arabic. There was an under-representation from low-income countries (LIC) with 36 participants (9.1%), and the rest of the participants were almost equally distributed between lower-middle-income countries (LMIC, *n* = 132, 33.5%), upper-middle income countries (UMIC, *n* = 111, 28.2%) and high-income countries (HIC, *n* = 115, 29.2%) (Table 1).

**Table 1:** Participant characteristics.

Professional group	n	%
Primary Care Provider	197	50.0
*General Practitioner or Family Doctor *	*181*	*91*.*9*
*Mid-level Health Care Provider *	*4*	*2*.*0*
*Registered Nurse or Nurse Practitioner *	*8*	*4*.*1*
*Other Type of Primary Care Provider *	*4*	*2*.*0*
Government Staff or Policymaker	31	7.9
Academic or Researcher	118	29.9
Other professional group, of which:	48	12.2
*Public Health*	*6*	*12*.*5*
*NGO*	*4*	*8*.*3*
*Student *	*27*	*56*.*3*
*Other *	*11*	*22*.*9*
**Gender**		
Male	183	46.4
Female	209	53.0
Gender diverse	2	0.5
**Language of survey completion**		
Arabic	0	0.0
English	306	77.7
Chinese	5	1.3
French	27	6.9
Portuguese	5	1.3
Spanish	51	12.9
Russian	0	0.0
**Country income classification quartiles**		
Low-income country	36	9.1
Lower-middle-income country	132	33.5
Upper-middle-income country	111	28.2
High-income country	115	29.2
	***Mean***	***Range***
**Years of professional experience**	14	0–48

NGO: Non-governmental organization.

### National COVID-19 vaccination coverage

**Fig 2:**
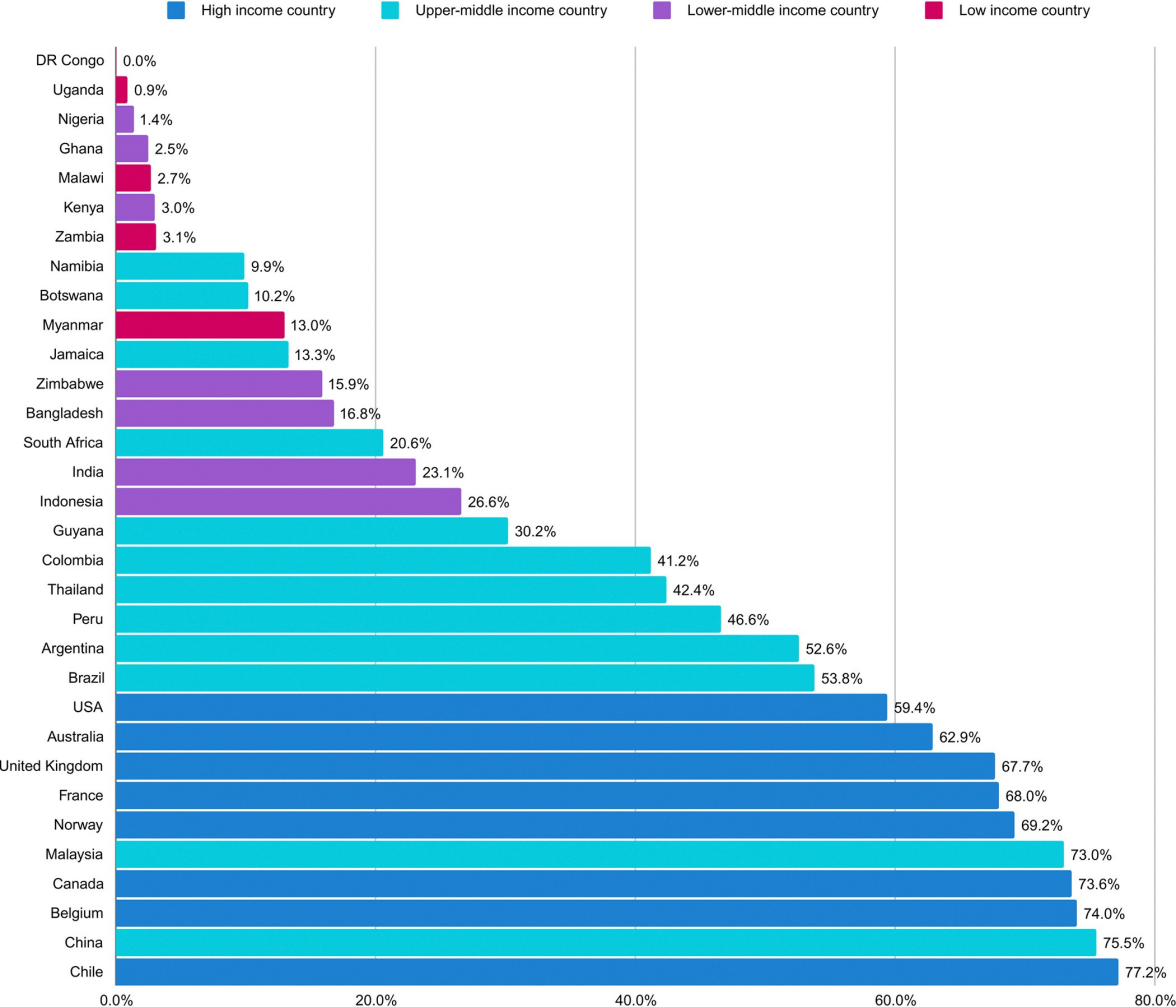
National COVID-19 vaccination coverage.

Amongst the 32 countries above the threshold response, the national COVID-19 vaccination coverage for completion of an initial vaccine regime ranged from 0.04% (Democratic Republic of Congo) to 77.23% (Chile) (Fig 2). The median national COVID-19 vaccination coverage across the 32 countries was 28.41% at the time of survey distribution.

#### Perceptions of effectiveness of COVID-19 vaccine programs

In general, perceived effectiveness of COVID-19 strategies decreased with decreasing economic quartiles (Table 2). Perceived PC contribution increased the higher the economic quartile. In our linear regression model, perceived effectiveness of COVID-19 vaccine programmes was significantly associated with national vaccination coverage (*ß* = 0.468, *P<0*.*001*), providing some internal validity to study results.

**Table 2:** Perceptions of effectiveness of COVID-19 vaccine programs by economic quartile.

	Not at all		Slightly		Moderately		Highly		Extremely		Don’t know	
	*n*	%	*n*	%	*n*	%	*n*	%	*n*	%	*n*	%
LICs (*n* = 36)	4	11.1	11	30.6	15	41.7	6	16.7	0	0.0	0	0.0
LMICs (*n* = 132)	4	3.0	11	8.3	48	36.4	53	40.2	14	10.6	2	1.5
UMICs (*n* = 111)	0	0.0	14	12.6	44	39.6	31	27.9	21	18.9	1	0.9
HICs (*n* = 115)	0	0.0	1	0.9	28	24.6	60	52.6	25	21.9	0	0.0

LICs: Low-income countries; LMICs: Lower-middle-income countries; UMICs: Upper-middle-income countries; HICs: High-income countries.

#### Perceptions of public health and primary care integration

The majority of participants, regardless of country income level, perceived that PC and PH were “moderately” working together effectively (LICs: 34.3%, LMICs: 31.1%, UMIC: 38.2%, HICs: 42.6%). However, the proportion of participants who perceived that PC and PH were “highly” or “extremely highly” working together effectively was greatest in LICs (34.3%) and continued to decrease as the income level increased (LMICs: 31%, UMICs: 21%, HICs: 15.6%). Perception of PC contributions to the COVID-19 vaccine programme was weakly positively correlated with perception of PC-PH integration (*r*_*s*_ = 0.31, *P*<0.001) across all countries.

**Fig 3:**
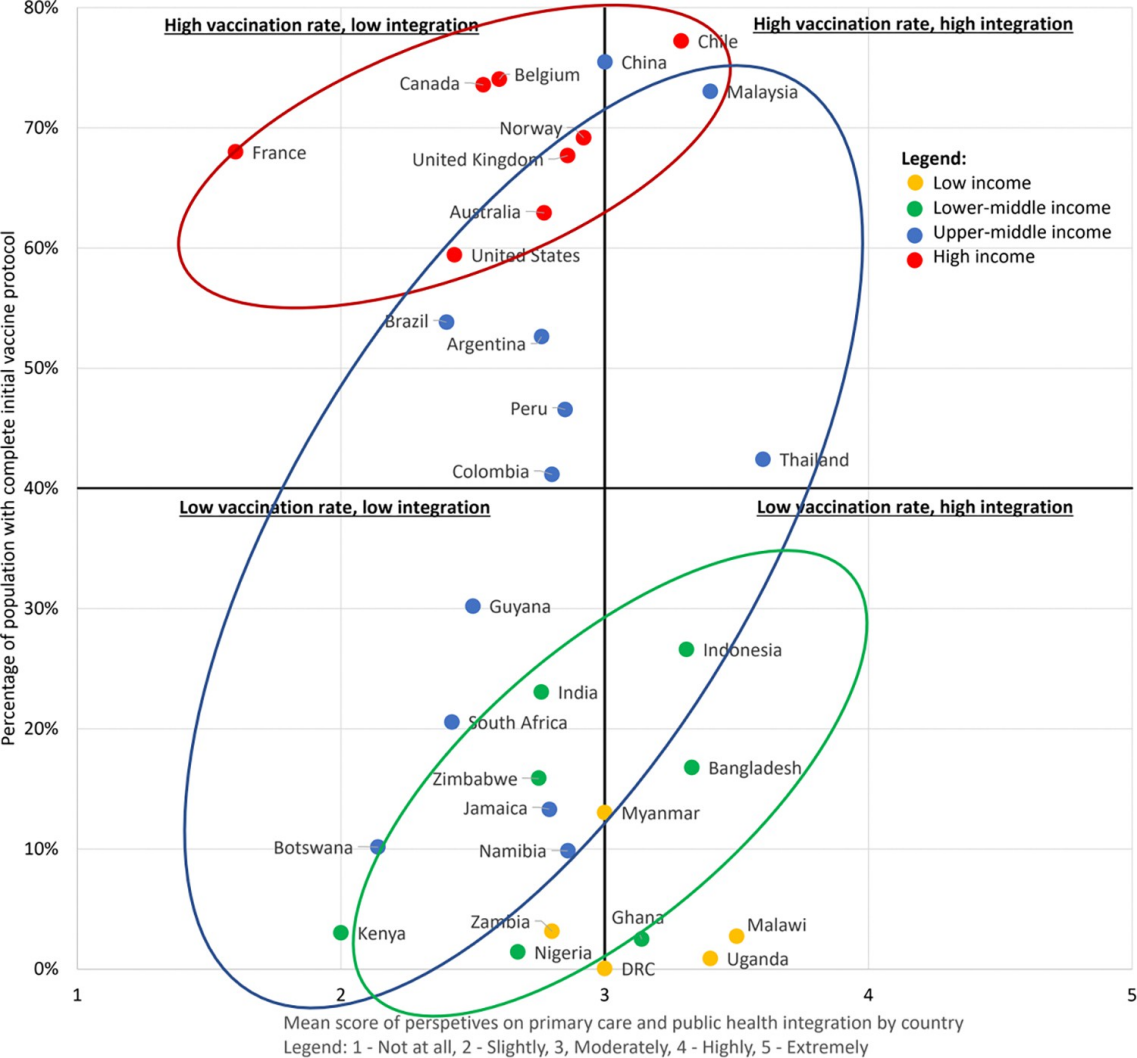
Association between perception of primary care-public health integration and national COVID-19 vaccination coverage.

We found a statistically significant association between perception of PC-PH integration and national vaccination coverage in LMIC (*ß* = 0.218, *P* = 0.014) and UMIC (*ß* = 0.195, *P* = 0.041) countries (Fig 3). A non-significant trend was observed in HICs and LICs could not be analysed due to insufficient data.

### Perceived barriers to national COVID-19 vaccination coverage

Across all countries, the top two negative correlations between perceived barriers and national vaccine coverage were patient hesitancy (*r*_*s*_ = -0.41, *P*<0.001), and lack of vaccine supply (*r*_*s*_ = -0.41, *P*<0.001). Additional barriers were lack of availability of trained vaccinators (*r*_*s*_ = -0.34, *P*<0.001); lack of adequate cold supply chain (*r*_*s*_ = -0.32, *P*<0.001), and lack of political commitment (*r*_*s*_ = -0.21, *P*<0.001).

### Perceived facilitators to national COVID-19 vaccination coverage

#### Vaccine products used

A diverse variety of vaccine products were used across all jurisdictions (Table 3). The majority of participants, regardless of income level, reported vaccines were available for free in the public sector (LICs 97.2%, LMICs, 96.2%, UMICs 100%, HICs 100%). Across all countries, utilisation of the Pfizer-BioNTech and Moderna vaccines was positively correlated with national vaccination coverage (*r*_*s*_ = 0.44, *P*<0.001 and *r*_*s*_ = 0.20, *P*<0.001 respectively).

**Table 3:** Use of different vaccine products among countries by economic quartile.

	LICs		LMICs		UMICs		HICs	
	(*n* = 36)		(*n* = 132)		(*n* = 111)		(*n* = 115)	
	*n*	%	*n*	%	*n*	%	*n*	%
Pfizer-BioNTech	17	47.2	41	31.1	105	94.6	115	100.0
Moderna	13	36.1	63	47.7	43	38.7	94	81.7
Vaxzevria by Oxford-AstraZeneca	21	58.3	40	30.3	73	65.8	64	55.7
Covishield by Serum Institute of India	10	27.8	37	28.0	31	27.9	2	1.7
Janssen (Johnson&Johnson)	22	61.1	29	22.0	61	55.0	31	27.0
Sputnik V by Gamaleya	2	5.6	22	16.7	30	27.0	3	2.6
Sinovac	12	33.3	65	49.2	40	36.0	21	18.3
Sinopharm	7	19.4	24	18.2	54	48.6	2	1.7
Covaxin by Bharat Biotech	5	13.9	29	22.0	3	2.7	0	0.0
Other, of which:	0	0.0	3	2.3	5	4.5	11	9.6
*CanSino*	*0*	*0*.*0*	*0*	*0*.*0*	*5*	*4*.*5*	*10*	*8*.*7*
*Novavax*	*0*	*0*.*0*	*1*	*0*.*8*	*0*	*0*.*0*	*1*	*0*.*9*
*Zydus Cadila*	*0*	*0*.*0*	*1*	*0*.*8*	*0*	*0*.*0*	*0*	*0*.*0*
*Zifivax*	*0*	*0*.*0*	*1*	*0*.*8*	*0*	*0*.*0*	*0*	*0*.*0*

#### Equity and access

Community engagement was perceived to be slightly-to-moderately low and appropriate use of health data to be moderate across all jurisdictions. Most respondents reported that vulnerable and high-risk groups (based on medical professional status, sociodemographic risks and other occupational risks) were prioritised. Across all countries, there was a very weak positive correlation between level of community engagement and national vaccine coverage (*r*_*s*_ = 0.12, *P* = 0.024), plus a weak positive correlation between prioritisation of vulnerable and high-risk groups and national vaccination coverage (*r*_*s*_ = 0.33, *P*<0.001).

## Discussion

### Summary

Our findings show that while overall there was significant spread and no significant difference in perceived PC-PH integration between all respondent countries, lower income economies were more likely to report stronger integration. Further, UMIC and LMIC showed a significant correlation with perceived integration with completion of initial vaccine regime, while HIC only showed a non-significant trend.

### Strengths and limitations

The strength of this research is capturing many different contexts from LICs to HICs. In addition, the study was planned with and international team representing each continent. The main limitation of our study is self-report bias due to convenience and snowball sampling, and relying on participants’ perceptions about past, even though recent events. Convenience sampling prevent the ability to comment on representativeness of responders, as well as the ability to report on all nations, thus, in an attempt to overcome these limitations, countries with <5 responses were excluded [20].

Self-reporting of being a primary care professional is also a limitation, as there are a multitude of primary care professional cadres across the different countries, however self-report can be a valid and reliable tool for self-identification and has been used effectively in previous studies on similar topics [20]. In addition, as a survey, not interview-based study, we were unable to verify respondents’ definitions of integration, which is another limitation, as this can vary in the literature. We also did not capture sufficient responses from LICs to permit statistical analysis in this economic quartile; this under-representation limits generalizability to that context.

### Comparison with existing literature

Our finding that PC-PH integration was an important factor in COVID-19 vaccination campaigns builds on previous work linking integration and mortality [20].

Historically, the involvement of PC in population-wide vaccination campaigns improves capture of key populations such as elderly or vaccine-hesitant patients [13,18]. Emerging studies confirm this during the COVID-19 pandemic [30], especially for key populations affected by high baseline inequity [31,32]. However, integration alone does not suffice, as resource availability is clearly the most significant confounder. At time of writing, only just over a third of individuals in LICs had received even one dose of any COVID-19 vaccine [3].

Doherty describes five levels of collaboration towards integration: minimal, from a distance, onsite (co-location), with some integration of services and systems, and full integration including influence-sharing [33]. The degree and type of integration required for a particular service will vary greatly depending on local realities and will have to be carefully planned to suit the context. Furthermore, as the WHO and others point out, PC-PH integration for the purpose of population-wide campaigns is not without risk. It has the potential logistical and financial challenges, and spillover of vaccine hesitancy into routine vaccination campaign, particularly in settings where the COVID-19 pandemic has led to an erosion of trust in medical institutions [8,34,35].

Few large, good quality, rigorously designed studies exist to guide PC-PH integration in general [36]. A study drawing lessons from COVID-19 vaccination campaigns of 11 African countries concluded that integration needs to be multi-sectoral (extending beyond the health system) and requires an intimate knowledge of local realities, constraints, as well as possible improvements to partnerships and infrastructure [37]. There has been wide diversity globally around the policy, coverage and demand for COVID-19 vaccines, which can affect integration efforts. During the COVID-19 pandemic, vaccine coverage was correlated with healthcare access and quality, sociodemographic profile, and gross domestic product of a country [38]. Wealthier nations benefited from early access to vaccines while low- and middle-income countries faced persistent shortages due to challenges with procurement and distribution [39]. The COVAX platform, a public-private partnership between the WHO, Gavi, the Vaccine Alliance, and the Coalition for Epidemic Preparedness Innovations, was established early in the COVID-19 pandemic to ensure access to COVID-19 vaccine doses (helping with procurement and distribution), and later in the pandemic in 2022, the Access to COVID-19 Tools (ACT) Accelerator was established as another private-public partnership to provide operational support for vaccination programs in countries with vaccination coverage below 10%, although gaps and weaknesses in funding, structures and processes have been described [40]. In some high income countries, partnerships between the public and private sector contributed substantially to the overall emergency response, although not in the vaccination or integration efforts [41]. More country-based case studies are emerging [42], mostly from MIC [35,42], and more are needed. We aim to further these studies from the outperformers emerging from our data (e.g. Chile, Malaysia, and Indonesia for HICs, UMICs and LMICs respectively) as we believe that there is a need for further research to establish causality and better explore mechanisms of PC-PH integration in multiple contexts.

### Implications for practice

The COVID-19 pandemic has underscored the critical role of integrated approaches in PH. In the context of vaccination campaigns, the involvement of PC has historically led to improved coverage, especially among key populations such as the elderly and vaccine-hesitant individuals. Emerging studies during the pandemic further support this, demonstrating not only benefits for specific populations but also for overall vaccination rates.

While there was significant variation in the perceived integration of PC and PH across different countries, lower-income economies tended to report stronger integration. Moreover, as we move down economic quartiles, the perception of "high" or "extremely high" integration increases, indicating that LIC placed a greater emphasis on this collaborative approach. Comparing perceived integration levels with actual vaccination rates at the time of the survey distribution, LMIC and UMIC exhibited the most significant correlation, likely because they had to seek efficiencies.

This suggests that PC-PH integration played a pivotal role in the success of COVID-19 vaccination campaigns and reinforces previous findings linking integration to positive health outcomes. Integration may be most important in middle-income economies that have just adequate supply of vaccine, whereas it may be superfluous in over-supply conditions in high-income economies, it is insufficient to overcome the lack of supply in low-income economies.

High-income economies have been on a path of specialisation, fragmentation and sometimes duplication that can hinder integration. While efforts at integrating health systems in these contexts will differ from those of lower-income economies, common lesson can be shared [43]. At the very least, the ongoing effects of the COVID-19 crisis including the system stoppages, the increasing cost of materials, and increasing inequity argue for more communication between primary care and public health towards a more concerted and rational approach to care delivery [44].

## Supporting information

S1 File FM vax: An international survey on the integration of public health and primary care in COVID-19 vaccination campaigns. (DOCX)

S2 File List of primary health care professional networks and member-based organizations targeted to help distribute the survey. (DOCX)
