# Supplementary material for: Does integration matter? an international cross-sectional study on the relationship between perceived public health and primary care integration and COVID-19 vaccination rates
Source: PLoS One. 2025 Feb 21;20(2):e0317970. doi: 10.1371/journal.pone.0317970 (PMC11845041; doi:10.1371/journal.pone.0317970)
Supplement: S1 File — (DOCX) [file pone.0317970.s001.docx]

S1 File. FM Vax: An International Survey on the Integration of Public Health and Primary Care in COVID-19 Vaccination Campaigns.

Sections

[Introduction 1](#_bookmark0)

[Part 1 of 3: General Information 2](#_bookmark1)

[Part 2 of 3: COVID-19 Vaccination Programme 3](#_bookmark2)

1. [Planning, coordination and service delivery 3](#_bookmark3)
2. [Perspectives on the implementation of the COVID-19 vaccination programme 4](#_bookmark4)
3. [Primary care involvement in the COVID-19 vaccination programme rollout 6](#_bookmark5)
4. [Suggestions on how to improve the rollout 6](#_bookmark6)

[Part 3 of 3: Public Health and Primary Care Integration 7](#_bookmark7)

1. [Primary care attributes 7](#_bookmark8)
2. [Perspectives on population health approach 8](#_bookmark9)

[Contact Information 9](#_bookmark10)

# Introduction

This survey is being conducted to better understand the primary health care approach used by different countries in the implementation of their COVID-19 vaccination programmes, with a focus on the integration of public health and primary care.

In this survey, please answer from the perspective that best describes the COVID-19 vaccination plan in **your context.**

Please click here to access definitions of key terms used in the survey.

*[The text box will appear in a new window]*

According to the World Health Organization, [**primary health care**](https://www.who.int/docs/default-source/primary-health/vision.pdf) is a society-wide approach that aims to ensure equitable access to the highest possible level of health by focusing on people's needs and preferences. The key components of primary health care are: (1) multi- sectoral policy and action, (2) empowered and engaged communities and (3) an integrated health services model that prioritizes **primary care** and essential **public health functions** to ensure effective care delivery.

[**Primary care**](https://www.who.int/docs/default-source/primary-health/vision.pdf) is a patient’s first point of contact in the healthcare system for accessible,

continued, comprehensive and coordinated patient-focused care.

**Primary care providers** are health professionals that provide primary care when a person needs to see or talk to someone about their health. This can include a family doctor or general practitioner, mid-level health provider, community pharmacist, nurse practitioner or community health worker.

[**Essential public health functions**](http://www.emro.who.int/about-who/public-health-functions/index.html) are the necessary collective actions, under the primary responsibility of the state, that are fundamental to improving, promoting, protecting and restoring the health of the population.

# Part 1 of 3: General Information

This section asks you to provide general information about yourself and the country, state or province that you work in and that you will be referring to in this survey.

**Q1.1**. Although you may identify with more than one of the professional groups below, please indicate the one that best describes your current role and perspective.

- Primary Care Provider
- Government Staff or Policymaker
- Academic or Researcher
- Other (please specify) [open text]

**Q1.2.** *[If “Primary Care Provider” is selected, the following question will appear]*

As a Primary Care Provider, please specify your professional title:

- General Practitioner or Family Doctor
- Mid-level Health Care Provider: e.g., Clinical Associate, Physician Assistant, Medical Assistant or Clinical Officer
- Registered Nurse or Nurse Practitioner
- Community Pharmacist
- Other Type of Primary Care Provider (please specify) [open text]

**Q2.** How many years of experience do you have in your field?

**Q3.** Please select the country where you work:

▼ Afghanistan ... Zimbabwe

**Q4.** Please specify the state or province where you work:

- Province/state [open text]
- Not applicable

**Q5.** What is your gender?

- Male
- Female
- Gender diverse

# Part 2 of 3: COVID-19 Vaccination Programme

This section is specific to the current COVID-19 vaccination programme in **your context.**

## Planning, coordination and service delivery

**Q6.** Which COVID-19 vaccines are currently in use? Select all that apply.

- Pfizer-BioNTech
- Moderna
- Vaxzevria by Oxford-AstraZeneca
- Covishield by Serum Institute of India
- Janssen (Johnson&Johnson)
- Sputnik V by Gamaleya
- Sinovac
- Sinopharm
- Covaxin by Bharat Biotech
- Other (please specify) [open text]
- Other (please specify) [open text]
- Other (please specify) [open text]
- Don’t know

**Q7.** What is the vaccine registration process? Select all that apply.

- Register for an appointment online through a government website
- Register for an appointment with a local health facility e.g., hospital, clinic, pharmacies
- No prior appointment needed, walk-in registration to get your vaccination
- Other (please specify) [open text]
- Don’t know

**Q8**. What type of locations are being used to vaccinate people? Select all that apply. Vaccination centres in:

- Public primary care facilities
- Private primary care facilities
- Public hospitals
- Private hospitals
- At non-health facilities e.g., churches/mosques, warehouses, sports arena, etc...
- Mobile clinics
- At home
- Other (please specify) [open text]
- Don’t know

**Q9.1** How does your health system track people that have been vaccinated? Select all that apply.

- Patient held paper-based immunization records/booklets
- Digital immunization certificate (through mobile or web-based apps)
- Regional or national electronic immunization registries
- Other (please specify) [open text]

**Q9.2**. *[The following question will only appear if “Regional or national immunization registries” is selected]* Can primary care providers access the immunization registries to track the vaccination status of their patients?

- Yes
- No
- Don’t know

**Q10.** How is the payment for COVID-19 vaccine arranged from the recipients’ perspective?

Select all that apply.

- Vaccination is free in public sector
- Vaccination requires a co-payment in public sector
- Vaccination is covered by health insurance in private sector
- Vaccination can be bought over the counter in private sector
- Other (please specify) [open text]

## Perspectives on the implementation of the COVID-19 vaccination programme

**Q11.** In your opinion, to what extent does the COVID-19 vaccination programme promote equitable access of the vaccine to the following priority groups?

|  | Not at  all | Slightly | Moderat  ely | Highly | Extrem  ely | Don’t  know |
| --- | --- | --- | --- | --- | --- | --- |
| **Medical priority groups** (e.g. elderly, immunocompromised, health comorbidities, long  term residential facility) |  |  |  |  |  |  |
| **Sociodemographic**  **priority groups**  (e.g. homeless, people living in extreme poverty or in urban slums, disadvantaged ethnic minorities, refugees,  prisoners, low-income migrant workers) |  |  |  |  |  |  |
| **Professional priority**  **groups** (e.g. healthcare |  |  |  |  |  |  |

| workers, teachers, police  officers, municipal services, agriculture and food workers, transportation workers, any other profession deemed  essential) |  |  |  |  |  |  |
| --- | --- | --- | --- | --- | --- | --- |

**Q12.** In your opinion, to what extent have the following major barriers been encountered during the rollout of the COVID-19 vaccination programme?

|  | Not at  all | Slightly | Moderat  ely | Highly | Extrem  ely | Don’t  know |
| --- | --- | --- | --- | --- | --- | --- |
| Unable to identify priority  groups |  |  |  |  |  |  |
| Unable to reach priority  groups |  |  |  |  |  |  |
| Patient refusal/hesitancy |  |  |  |  |  |  |
| Lack of availability of cold  or ultra-cold supply chains |  |  |  |  |  |  |
| Not enough trained  vaccinators |  |  |  |  |  |  |
| Not enough vaccines |  |  |  |  |  |  |
| Lack of political  commitment |  |  |  |  |  |  |
| Lack of clear vaccine  policies |  |  |  |  |  |  |
| Other barriers (please  specify) [open text] |  |  |  |  |  |  |
| Other barriers (please  specify) [open text] |  |  |  |  |  |  |
| Other barrier (please  specify) [open text] |  |  |  |  |  |  |

**Q13.** In your opinion, how effective have the current vaccine strategies been during the COVID- 19 vaccine programme rollout?

- Not at all
- Slightly
- Moderately
- Highly
- Extremely
- Don’t know

## Primary care involvement in the COVID-19 vaccination programme rollout

**Q14.** How are primary care providers involved in the COVID-19 vaccination programme rollout? Select all that apply.

- Vaccinating in their usual practice settings
- Vaccinating outside their usual practice settings, but in the same community
- Vaccinating outside their usual practice setting and community
- Not involved
- Other (please specify) [open text]
- Don’t know

**Q15.** In your opinion, to what extent has primary care contributed to the implementation of the COVID-19 vaccination programme rollout?

- Not at all
- Slightly
- Moderately
- Highly
- Extremely
- Don’t know

## Suggestions on how to improve the rollout

**Q16.** What best practices can be identified from the implementation of the COVID-19 vaccination?

- [Open text]

**Q17.** What is the number one thing you would do differently or change about the COVID-19 vaccination programme going forward?

- [Open text]

# Part 3 of 3: Public Health and Primary Care Integration

This final section assesses your general perception of public health and primary care integration**.** Please take into account all elements of the health systems available in your context, both the public and private sectors.

## Primary care attributes

**Q18**. Do patients hold a unique identifier used regionally or nationally across health care settings (e.g., a Unique Patient Identification Number or UPIN)?

- Yes, for the entire population
- Yes, but only for segments of the population
- No
- Don’t know

**Q19.** How are patients assigned to a primary care facility?

- Geographically: patients must live in a defined geographic area served by the facility
- List of patients only: patients must be registered with the facility, but can come from anywhere
- Combined: patients must be registered with the facility and live in the defined geographic area served by the facility
- No coordinated strategy: patients can go anywhere that they choose
- Other (please specify) [open text]
- Don’t know

**Q20.** How are primary care providers involved in providing care to the community? (This could include community health workers as well as any other primary care providers)

Select all that apply.

Primary care providers:

- Conduct home visits to people with specific health needs (e.g., impairments, terminally ill, pregnant women)
- Conduct home visits to treat people with acute problems (e.g., pneumonia, malaria, diarrhea)
- Conduct home visits to follow-up patients with chronic diseases (e.g., HIV, TB, non- communicable diseases)
- Conduct home visits for early access to preventive services (e.g., vaccinations, cancer screening, family planning provision) or to provide health education .
- Facilitate support for other groups in the community (e.g., for chronic conditions)
- Visit children in educational facilities (e.g., pre-school, school)
- Visit people in their workplace
- Visit people in residential care (e.g., elderly)
- Not applicable, primary care providers only see patients in healthcare facilities
- Other (please specify) [open text]
- Don’t know

**Q21.** Is a referral from primary care providers required to obtain care from a specialist or hospital (gatekeeper role) except in cases of emergency?

- Yes
- Sometimes
- No
- Don’t know

**Q22.** Do primary care providers perform routine vaccinations in their own practice settings (e.g., influenza or HPV vaccines)?

- Never
- Rarely
- Sometimes
- Often
- Always
- Don’t know

## Perspectives on population health approach

**Q23.** In your opinion, to what extent does your health system engage with members of the community to identify their health needs or to improve services?

- Not at all
- Slightly
- Moderately
- Highly
- Extremely
- Don’t know

**Q24.** In your opinion, to what extent are local health care priorities informed by robust data on the needs of the community?

- Not at all
- Slightly
- Moderately
- Highly
- Extremely
- Don’t know

**Q25.** In your opinion, to what extent are the people responsible for public health and the people responsible for primary care working closely together?

- Not at all
- Slightly
- Moderately
- Highly
- Extremely
- Don’t know

# 
