# Supplementary material for: Does integration matter? an international cross-sectional study on the relationship between perceived public health and primary care integration and COVID-19 vaccination rates
Source: PLoS One. 2025 Feb 21;20(2):e0317970. doi: 10.1371/journal.pone.0317970 (PMC11845041; doi:10.1371/journal.pone.0317970)
Supplement: S2 File — (DOCX) [file pone.0317970.s002.docx]

S2 File. List of Primary health care professional networks and member-based organizations targeted to help distribute the survey.

1. Afro PHC
2. Ariadne Labs
3. Asociacion Metropolitana de Medicina Familiar
4. Australasian Association for Academic Primary Care
5. College of Family Physicians of Canada
6. Dirección de Formación y Educación Permanente Ministerio de Salud de la Prov de Buenos Aires
7. European Forum for Primary Care
8. European General Practice Research Network
9. Family Physician Forum for Cape Town
10. Federacion Argentina de Medicina Familiar y General
11. George Institute for Global Health
12. Institute of Disaster Preparedness and Response in Hong Kong
13. International Council of Nurses
14. North American Primary Care Research Group
15. Primafamed
16. Primary Health Care Research Consortium
17. Robert Graham Center
18. South African Pharmacy Council
19. The George Institute, Australia
20. The George Institute, India
21. The South African Academy of Family Physicians
22. Universidad Nacional del Sur
23. World Organization of Family Doctors (WONCA)
24. WONCA – Working Party on Research
25. WONCA - Young Doctors
